# Evaluation of seasonal patterns of Kawasaki Syndrome- and rotavirus-associated hospitalizations in California and New York, 2000-2005

**DOI:** 10.1186/1471-2431-9-65

**Published:** 2009-10-16

**Authors:** Adam MacNeil, Robert C Holman, Krista L Yorita, Claudia A Steiner, Umesh D Parashar, Ermias D Belay

**Affiliations:** 1 Division of Viral and Rickettsial Diseases, National Center for Zoonotic, Vector-borne and Enteric Diseases, Centers for Disease Control and Prevention, US Department of Health and Human Services, Atlanta, GA, USA; 2 Healthcare Cost and Utilization Project, Center for Delivery, Organization and Markets, Agency for Healthcare Research and Quality, US Department of Health and Human Services, Rockville, MD, USA; 3 Division of Viral Diseases, National Center for Immunization and Respiratory Diseases, Centers for Disease Control and Prevention, US Department of Health and Human Services, Atlanta, GA, USA

## Abstract

**Background:**

Kawasaki Syndrome (KS) is an uncommon childhood disease with unknown etiology. It has been suggested that rotavirus infection may play a causative role in the development of KS.

**Methods:**

To examine potential temporal associations between KS and rotavirus infection, seasonal patterns of KS- and rotavirus-associated hospitalizations among children in California and New York during 2000-2005 were compared.

**Results:**

Rotavirus hospital admissions were markedly winter seasonal, with very few summer hospitalizations. KS hospitalizations occurred year-round but also peaked slightly during winter and spring.

**Conclusion:**

The strong winter seasonal pattern of rotavirus clearly differed from the year-round pattern of KS hospitalizations. While the present study cannot completely rule out rotavirus as having a role in the development of KS, other agents must be involved in the etiology of KS.

## Background

In February 2006, a new vaccine against rotavirus, RotaTeq, was licensed by the US Food and Drug Administration, and currently the Advisory Committee on Immunization Practices recommends routine immunization of all US children against rotavirus, starting at 6 weeks of age [1]. In June 2007, the FDA revised the package label for RotaTeq to include information on cases of Kawasaki syndrome (KS), due to rare reported cases in pre-licensure studies [2]. Based on a review of pre-licensure and available post-licensure surveillance data, the FDA concluded that there is not a known cause and effect relationship between receiving RotaTeq and the occurrence of KS.

KS is an uncommon childhood illness that is characterized by high fever and inflammation of the blood vessels throughout the body. Approximately 4,000 children each year in the United States develop KS, about eighty percent of whom are younger than 5 years of age [3,4]. The cause of KS is unknown [5]; it has been hypothesized that an infectious agent is involved in the etiology of KS [6] but no specific association has been confirmed. In a single study from Japan, where a higher incidence of KS than in the United States has been noted, rotavirus particles or capsomers were identified in 74% of 39 KS cases versus 11% of 18 controls and a rise in serum antibody to rotavirus was identified in 51% of 75 KS cases versus 8% of 39 controls [7]. While these data raised the possibility of an association between rotavirus infection and KS, no studies to confirm these findings have been reported.

Because the etiology of KS remains unknown, and the possibility of an association between KS and rotavirus infection has been raised [7], we sought to further examine the relationship between these diseases. Furthermore, a better understanding of any potential association between KS and natural rotavirus infection could help assess the biologic plausibility of a relationship between rotavirus vaccination and KS. Therefore, we examined the seasonal patterns of hospitalizations associated with rotavirus gastroenteritis and with KS for the US states of California and New York, to evaluate whether the two diseases temporally coincided.

## Methods

Hospital discharge records for the period from 2000 through 2005 for California and New York residents <5 years of age with KS or with rotavirus listed as a diagnosis were analyzed from the State Inpatient Database (SID) in collaboration with the Healthcare Cost and Utilization Project (HCUP), Agency for Healthcare Research and Quality [8]. The states partner with the HCUP to produce the SID. The SID includes of all hospital discharge records from all community hospitals in the two states. Because HCUP data is secondary administrative data without clear patient identifiers, it has been determined that the data is exempt from ethical approval.

The International Classification of Diseases, 9^th ^revision, Clinical Modification (ICD-9-CM) [9] code 446.1 was used to identify KS-associated hospitalizations, and the code 008.61 was used to identify rotavirus-associated hospitalizations, listed as one of up to 15 or 25 diagnoses on the discharge record for New York and California, respectively. The average annual hospitalization rates (per 100000 children <5 years of age) were calculated by using the number of hospitalizations and the corresponding state census population for 2000 through 2005 [10]. For comparing the seasonal occurrence of KS and rotavirus, the hospitalization rates associated with KS and rotavirus for each state were plotted in comparison to the year and month of admission. To further assess whether hospitalizations due to KS and rotavirus coincide in a temporal manner, we determined the cumulative monthly proportion of hospitalizations during the 6-year study period for each of these diseases.

## Results

From 2000 through 2005, the annual average rates of hospitalization among children <5 years of age associated with KS and with rotavirus were 20.7 and 100.7 (per 100,000) in California, respectively, and 23.0 and 112.1 in New York, respectively.

**Figure 1:**
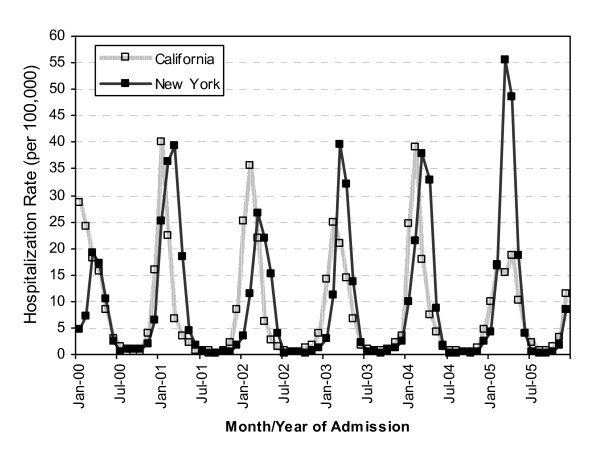
**Rotavirus-associated hospitalization rates by month of admission for children <5 years of age in California and New York, 2000-2005**.

**Figure 2:**
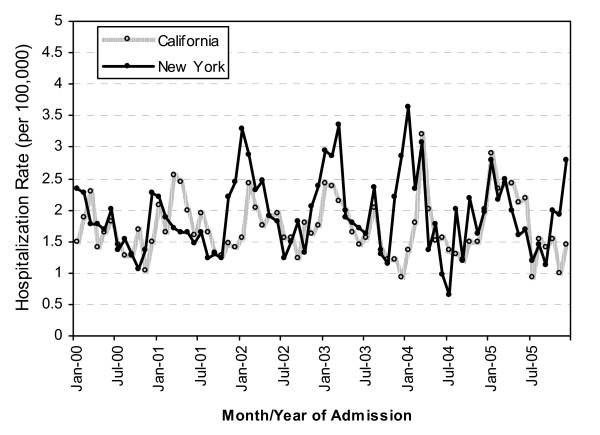
**Kawasaki syndrome (KS)-associated hospitalization rates by month of admission for children <5 years of age in California and New York, 2000-2005**.

During the study period, the rate of rotavirus-associated hospitalizations displayed a characteristic winter seasonal peak with very few cases reported during the summer months (Figure 1). With the exception of 2005, the rotavirus hospitalization rate peaked earlier in California than that in New York, with 1-2 months separating the seasonal peaks in the two states. KS-associated hospitalizations occurred year round, but the rate also peaked during the winter months (figure 2); however, the pattern displayed variability from year to year. During most years, the winter peaks of the KS hospitalization rate occurred earlier in New York than those in California.

**Figure 3:**
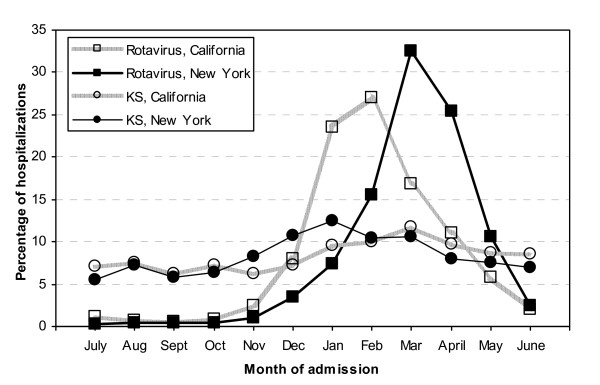
**Cumulative monthly proportion of overall rotavirus- and overall KS-associated hospitalizations, for children <5 years of age in California and New York, 2000-2005**.

To further compare the seasonal pattern of hospitalizations associated with rotavirus and with KS, we examined the composite proportion of hospitalizations by month of admission over the 6-year study period (figure 3). Rotavirus-associated hospitalizations clearly displayed a strong peak in the winter months, and this peak occurred about 1-2 months earlier in California than that in New York. In contrast, the occurrence of KS-associated admissions displayed a smaller peak in the winter and spring months and the timing of the peaks did not differ substantially between California and New York.

## Discussion

In both California and New York, during 2000-2005, rotavirus-associated hospitalization rates showed a strong winter seasonality and were nearly absent during the summer months, whereas KS-associated hospitalization rates occurred year round with smaller peaks during the winter months. Furthermore, the well recognized 1-2 month delay in winter peaks of rotavirus hospitalizations in the Northeast United States compared to winter peak the West United States [11] was not observed for KS-associated hospitalizations. Together, this does not support a strong temporal relationship between these diseases, and implies that other factors are involved in the etiology of KS. While the etiology of KS is still not understood, genetic factors, as well as other infectious agents may have a causative role.

It should be noted that although hospital discharge data are very useful in understanding disease occurrence, limitations in this methodology exist, for instance, not all rotavirus infections result in hospitalization. In addition, studies of this nature do not allow for assessment of individual-level associations between etiologic agents. Further discussions of limitations regarding this data source for KS and rotavirus, have been described elsewhere [3,4,12]. Finally, discharge data for this study was from all community hospitals in CA and NY. Community hospitals represent approximately 85% of all U.S. hospitals, including public and private, academic and specialty hospitals. In California, 98% of all community hospitals are included in the HCUP data base, and in New York, 100% of all community hospitals are included. While we believe this to be representative of discharge for all types of hospitals, we do not have comparison data which would allow us to make a definitive assessment.

## Conclusion

The occurrence of KS year-round indicates that etiologies other than rotavirus are involved in the occurrence of KS. Carefully designed prospective studies with microbiologic evaluation of clinical specimens from KS cases and an appropriate comparison group with similar age, sex, and genetic background are necessary to fully evaluate any possible causal relationship between KS and infection with rotavirus.

## Competing interests

The authors declare that they have no competing interests.

## Authors' contributions

AM participated in the design of the study and helped draft the manuscript. RH conceived of the study, participated in the design of the study, and participated in the statistical analysis. KY participated in the statistical analysis. CS coordinated the data collection and participated in the statistical analysis. UP conceived of the study, participated in the design of the study, and helped draft the manuscript. EB conceived of the study, participated in the design of the study, and helped draft the manuscript. All authors read and approved the final manuscript.

## Pre-publication history

The pre-publication history for this paper can be accessed here:



## References

[B1] Centers for Disease Control and Prevention (2009). Recommended Immunization Schedules for Persons Aged 0 Through 18 Years --- United States, 2009. MMWR.

[B2] Product Approval Information (FDA website). http://www.fda.gov/BiologicsBloodVaccines/Vaccines/ApprovedProducts/ucm142393.htm.

[B3] Holman RC, Curns AT, Belay ED, Steiner CA, Schonberger LB (2003). Kawasaki syndrome hospitalizations in the USA, 1997 and 2000. Pediatrics.

[B4] Holman RC, Shahriari A, Effler PV, Belay ED, Schonberger LB (2000). Kawasaki syndrome hospitalizations among children in Hawaii and Connecticut. Arch Pediatr Adolesc Med.

[B5] Rowley AH (2006). Finding the cause of kawasaki disease: a pediatric infectious diseases research priority. The Journal of infectious diseases.

[B6] Wang CL, Wu YT, Liu CA, Kuo HC, Yang KD (2005). Kawasaki disease: infection, immunity and genetics. The Pediatric infectious disease journal.

[B7] Matsuno S, Utagawa E, Sugiura A (1983). Association of rotavirus infection with Kawasaki syndrome. The Journal of infectious diseases.

[B8] HCUP SID Database Documentation [Healthcare Cost and Utilization Project (HCUP)]. http://www.ahrq.gov/data/hcup/.

[B9] Public Health Service and Health Care Financing Administration (2005). International classification of diseases 9th revision, clinical modification.

[B10] U.S. Bureau of the Census (2006). Intercensal estimates of the population of states by age, sex, and race: 2000-2005.

[B11] Torok TJ, Kilgore PE, Clarke MJ, Holman RC, Bresee JS, Glass RI (1997). Visualizing geographic and temporal trends in rotavirus activity in the United States, 1991 to 1996. National Respiratory and Enteric Virus Surveillance System Collaborating Laboratories. The Pediatric infectious disease journal.

[B12] Charles MD, Holman RC, Curns AT, Parashar UD, Glass RI, Bresee JS (2006). Hospitalizations Associated With Rotavirus Gastroenteritis in the United States, 1993-2002. The Pediatric infectious disease journal.

